# Microwave-Assisted Extraction of Phenolic Compounds from *Melastoma sanguineum* Fruit: Optimization and Identification

**DOI:** 10.3390/molecules23102498

**Published:** 2018-09-29

**Authors:** Cai-Ning Zhao, Jiao-Jiao Zhang, Ya Li, Xiao Meng, Hua-Bin Li

**Affiliations:** 1 Guangdong Provincial Key Laboratory of Food, Nutrition and Health, Guangdong Engineering Technology Research Center of Nutrition Translation, Department of Nutrition, School of Public Health, Sun Yat-Sen University, Guangzhou 510080, China; zhaocn@mail2.sysu.edu.cn (C.-N.Z.); zhangjj46@mail2.sysu.edu.cn (J.-J.Z.); liya28@mail2.sysu.edu.cn (Y.L.); mengx7@mail2.sysu.edu.cn (X.M.); 2 South China Sea Bioresource Exploitation and Utilization Collaborative Innovation Center, Sun Yat-Sen University, Guangzhou 510006, China

**Keywords:** *Melastoma sanguineum*, phenolic compounds, microwave-assisted extraction, response surface methodology, green extraction

## Abstract

A microwave-assisted extraction (MAE) technology optimized by response surface methodology (RSM) was established to extract phenolic compounds from the fruit of *Melastoma sanguineum*. The effects of solvent composition, ratio of solvent to material, temperature, time and microwave power on phenol yield were evaluated in single-factor tests. The three parameters exerting main impacts on phenol yield were further optimized by RSM. Under optimal extraction conditions (31.33% ethanol, solvent/material ratio of 32.21 mL/g, 52.24 °C, 45 min and 500 W), the total phenolic content was 39.02 ± 0.73 mg gallic acid equivalent (GAE)/g dry weight (DW). This MAE method performed better in comparison with two conventional methods, those being maceration (25.79 ± 1.03 mg GAE/g DW) and Soxhlet extraction (18.40 ± 1.34 mg GAE/g DW), using lower process temperature, shorter irradiation time, and lower organic solvent consumption. In addition, five flavonoids (epicatechin gallate, epicatechin, rutin, pigallocatechin and quercetin) and two phenolic acids (protocatechuic acid and chlorogenic acid) in the extract were identified and quantified using UPLC-MS/MS.

## 1. Introduction

The excessive generation of free radicals could oxidize intracellular macromolecules such as DNA, proteins and lipids, induce several diseases, and threaten human health [1,2]. Many studies have indicated that fruits are rich in antioxidants and possess extraordinary free radical scavenging properties, exhibiting a wide range of health benefits [3,4,5,6]. On account of this, there are long-standing interests in natural antioxidants, especially phenolic compounds [7,8,9]. 

*Melastoma sanguineum* (*M. sanguineum*) is a small shrub widely planted in Southeast Asia due to its attractive appearance and ornamental value. The fruit of *M. sanguineum* is edible, tasty, and can help digestion as a folk medicine [10]. Our previous research has found that *M. sanguineum* fruit possessed strong antioxidant capacity and was a good source of natural phenols [11]. Besides, an in-vitro study reported that some flavonoids in *M. sanguineum* fruit could contribute to preventing diabetic complications [12]. Owing to the potential health benefits, it is essential to extract and identify the phenolic compounds of *M. sanguineum* fruit, which will be helpful to investigate their bioactivities. 

The conventional extraction methods of phenolic compounds in plants are mainly maceration and Soxhlet extraction; disadvantages include high time-, energy- and solvent-consumption, and generating relatively low yields [13,14]. Hence, the usage of modern extraction technologies and the optimization of process parameters are necessary, and can effectively improve extraction efficiency. Nowadays, there are several new extraction methods including ultrasound-assisted extraction [15,16], supercritical fluid extraction [17], pressurized liquid extraction [18], as well as microwave-assisted extraction (MAE) [19]. The MAE is an efficient and environmentally friendly technology which has been used for the extraction of some phytochemicals from complex matrices like plants [20]. Microwave energy could rapidly increase the temperature inside plant cells through ionic conduction and dipole rotation, resulting in the rupture of cell walls, and accelerate the release of compounds into solvent [21]. Different from conventional extraction techniques, MAE requires a shorter time, less energy and less organic solvent consumption to produce higher yield [22]. Furthermore, MAE is easy-operating and economical, and therefore it is feasible to be applied in large-scale industrial production [23]. 

Several parameters could affect the extraction efficacy of MAE, such as solvent composition, solvent/material ratio, process temperature and duration, and microwave power [24]. The optimization of process parameters is crucial in warranting that the phenolic compounds could be extracted to the maximum extent. Response surface methodology (RSM) was employed to build the model, to investigate the effects of process parameters and their interactions on the response value, that is, the total phenolic content (TPC), and to optimize the extraction conditions. RSM is a mathematical statistical tool specified in modeling and optimizing technological parameters in multi-factor experimental design, with the purpose of maximizing the response value using the fewest experimental points [25]. In this study, dominant experimental parameters and their initial ranges were firstly determined through single-factor tests, and then optimized by RSM using a three-variable–five-level central composite design (CCD) methodology. Also, yields of MAE, maceration extraction and Soxhlet extraction were compared in terms of total phenolic and flavonoid contents, and antioxidant capacity of extracts. In addition, the phenolic profiles in the extracts of *M. sanguineum* fruits were identified and quantified by UPLC-MS/MS.

## 2. Results and Discussion

### 2.1. Results of Single-Factor Tests

**Figure 1:**
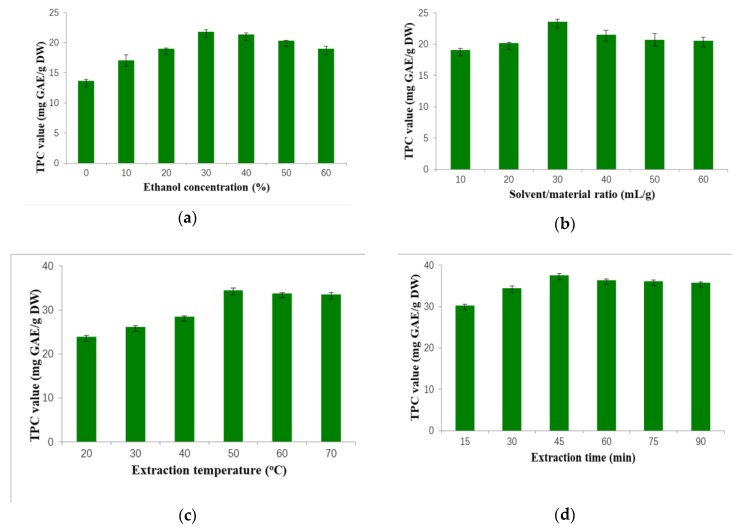

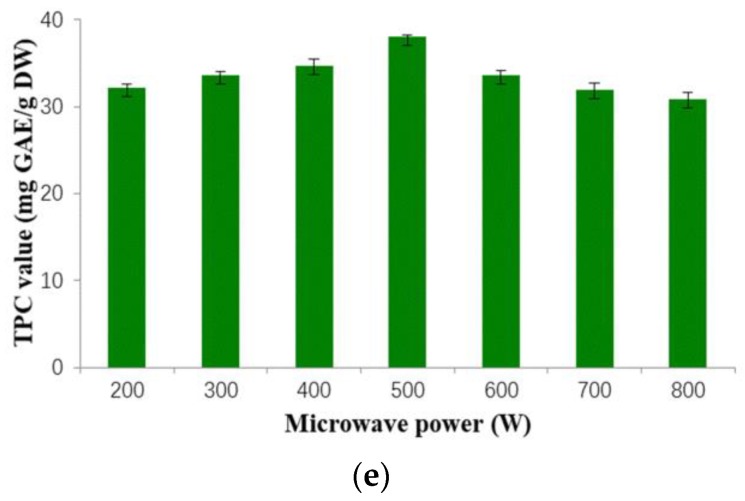
Effects of different factors on total phenolic content value of extracts (mg GAE/g DW): ethanol concentration (%) (**a**); solvent/material ratio (mL/g) (**b**); extraction temperature (°C) (**c**); extraction temperature (min) (**d**); and microwave power (W) (**e**). TPC: total phenolic content; GAE: gallic acid equivalent; DW: dry weight.

The solvent constitution decides the type and quantity of phenolic compounds extracted from plant materials, and is one of the most important factors in an extraction process [26]. Aqueous ethanol solution was widely utilized because it has low toxicity and good accessibility, and can easily dissolve phenolic compounds [27,28]. The concentration of ethanol could influence the polarity of solvent, which was critical for the solubility of phenolic compounds [24]. In this part, the effect of different concentrations of ethanol on phenol yield was analyzed, and other conditions remained constant as follows: 20 mL/g, 30 min, 30 °C and 500 W. As shown in Figure 1a, as the proportion of ethanol in hydroalcoholic solvent increased from 0% to 30%, the TPC value was improved significantly from 13.67 ± 0.25 to 21.81 ± 0.41 mg gallic acid equivalent (GAE)/g dry weight (DW). However, the TPC value gradually decreased when the concentration of ethanol continued to rise. Therefore, the 30% ethanol was considered proper for further experiments. 

Relatively high solvent volume could accelerate substance transfer and promote solubility, and then improve the extraction efficacy within a certain range [29]. The impact of solvent/material ratio (S/M ratio) on phenol yield was investigated from 10 to 60 mL/g under certain conditions (30% ethanol, 30 min, 30 °C, 500 W). Figure 1b shows that the TPC value increased from 10 to 30 mL/g, and reached the peak (23.57 ± 0.48 mg GAE/g DW) at 30 mL/g, then descended (40 mL/g), and maintained almost constant (40–60 mL/g). We speculated that when the TPC value reached the peak at 30 mL/g, the substance transfer probably reached the equilibrium. 

High temperature could speed up intermolecular interactions and facilitate molecular motion, which could increase the solubility of solute into the solution [24]. The effect of extraction temperatures was investigated when the other factors were kept constant (30% ethanol, 30 mL/g, 30 min, 500 W). When the temperature increased (20–50 °C), the TPC value rose remarkably from 23.88 ± 0.33 to 34.46 ± 0.74 mg GAE/g DW, and kept almost constant as further heating to 70 °C (Figure 1c). Thus, the optimum temperature (50 °C) was chosen in the next experiments. 

Figure 1d showed the influence of different extraction times on the TPC value when other conditions were fixed as: 30% ethanol, 30 mL/g, 50 °C and 500 W. Firstly, the TPC value increased from 30.26 ± 0.38 to 37.60 ± 0.43 mg GAE/g DW with duration increasing from 15 to 45 min. When duration was extended to 60 min, the extraction efficacy decreased, and kept constant thereafter. Possibly, long microwave irradiation could cause the degradation of some phenolic compounds [24]. Thus, the irradiation duration of 45 min was optimized for the next experiments. 

A sample of 0.500 g was extracted by 15 mL of 30% ethanol at 50 °C for 45 min, with different levels of microwave power. As shown in Figure 1e, the TPC value was improved with the increase of microwave power from 200 to 500 W, with a maximum of 38.08 ± 0.20 mg GAE/g DW at 500 W. As the irradiation power continued to rise, the TPC value descended gradually, which was possibly because excessive microwave power (>500 W) could cause the degradation of phenolic compounds. Hence, 500 W was chosen as the most efficient microwave power.

### 2.2. Results of Response Surface Methodology Optimization 

#### 2.2.1. Results of Central Composite Design 

In view of the above results, the three independent variables that exerted a dominant influence on phenol yield were selected in the CCD for further optimization. The coded levels (0, ±1, and ±1.68) and the corresponding actual levels of three independent variables are given in Table 1. 

#### 2.2.2. Model Fitting

The actual TPC values of 20 experimental combinations were fitted to a second-order polynomial model (Y—TPC value, X_1_—ethanol concentration, X_2_—S/M ratio, X_3_—temperature) with nonsignificant items being removed:

Y = −182.44 + 2.36X_1_ + 3.67X_2_ + 4.81X_3_ − 0.0164X_2_X_3_ − 0.0343X_1_^2^ − 0.0424X_2_^2^ − 0.0403X_3_^2^. (1)

The ANOVA in Table 2 declared that the fitted model was significant (*F* = 62.17, *p* < 0.0001). The nonsignificance of the lack-of-fit test (*p* = 0.0778) verified the suitability of the selected model. The R^2^ of 0.9824 implied that 98.24% of the variations of TPC value were attributed to the three independent variables. Besides, the adjusted R^2^ value of 0.9666 was close to the R^2^ of 0.9824, indicating that the observed values were correlated with the predicted values to a high degree. All the above results revealed the validity of the model to predict the real correlations between the response value and independent variables.

#### 2.2.3. Graphical Analysis

**Figure 2:**
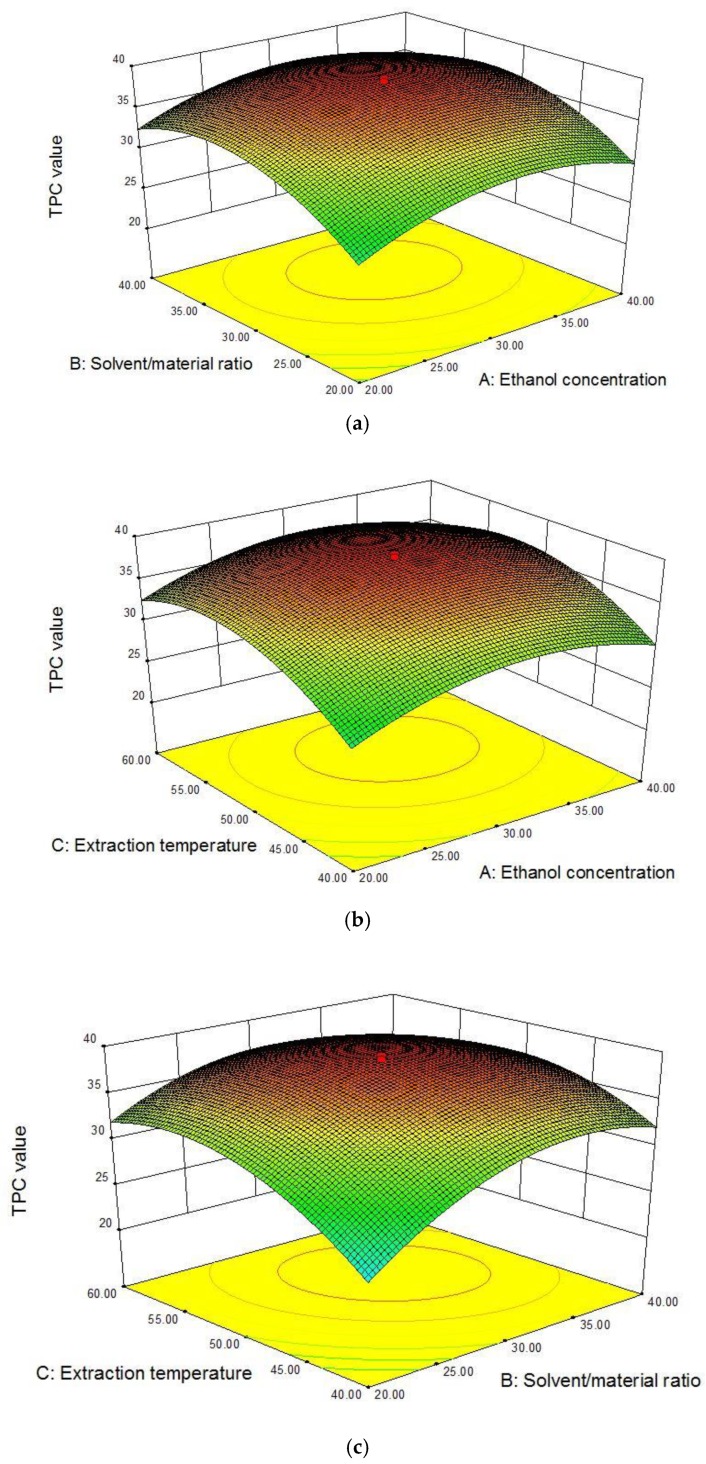
Graphical analysis of the effects of ethanol concentration (X_1_, %) and solvent/material ratio (X_2_, mL/g) (**a**); ethanol concentration (X_1_, %) and extraction temperature (X_3_, °C) (**b**); and solvent/material ratio (X_2_, mL/g) and extraction temperature (X_3_, °C) (**c**) on total phenolic content value (mg GAE/g DW).

The interaction between ethanol concentration and the S/M ratio with the TPC value is plotted in Figure 2a. The increase in ethanol concentration or S/M ratio obviously elevated the TPC value, and the TPC value reached the peak with about 30% ethanol and 30 mL/g. Figure 2b shows the interaction between ethanol concentration and temperature on the TPC value. The effect of ethanol concentration on the TPC value was similar to that in Figure 2a. With the extraction temperature increased from 40 to 50 °C, the TPC value was improved markedly, then decreased as the temperature continued to increase. Figure 2c plotted the interaction of S/M ratio and temperature with the TPC value, which was similar to those in Figure 2a,b. In view of the results above, the influence of three independent variables on the TPC value was not simply linear, and there were interactions between them. Given results of response surfaces plots and the ANOVA in Table 2, it could be concluded that all the three factors, concentration of ethanol, S/M ratio and temperature, significantly affected the response value.

#### 2.2.4. Verification of the Model

The optimal process parameters were as follows: 31.33% ethanol, 32.21 mL/g, 52.24 °C, 45 min and 500 W. Under the optimal extraction conditions, the actual data of 39.02 ± 0.73 mg GAE/g DW was comparable with the predicted value (39.24 mg GAE/g DW), which verified the accuracy of the model to predict the TPC yield. In addition, the TPC value was increased to 39.02 ± 0.73 mg GAE/g DW (after optimization) from 13.67 ± 0.25 mg GAE/g DW (before optimization), which indicated that the optimizing process was necessary. 

### 2.3. Comparison of MAE with Maceration and Soxhlet Extraction

The efficacy of MAE was compared with maceration and Soxhlet extraction in several aspects, such as duration, temperature, TPC, Trolox equivalent antioxidant capacity (TEAC), and total flavonoid content (TFC) values of extract (Table 3). The efficient MAE process increased the total phenol yield by 51.30% and 112.07% in comparison with maceration and Soxhlet extraction, respectively. In terms of the extraction duration, the best results were also acquired by MAE with only 45 min. Moreover, MAE cost less solvent and required lower temperature than those needed in the Soxhlet extraction. Furthermore, higher TEAC and TFC of the extract were obtained by the MAE method compared to the other two conventional approaches, further confirming its high efficiency. Similar results were also reported in comparing MAE with conventional extraction techniques in extracting polyphenols from *Gordonia axillaris* fruit [19], *Anoectochilus roxburghii* [30], and *Pistacia lentiscus* leaves [22].

### 2.4. Qualitative and Quantitative Measurement of Phenolic Compounds by UPLC-MS/MS

Due to the diversity of chemical structure, natural phenolic compounds present differently in pharmacological activities. Thus, identifying the type and content of phenolic compounds is essential to analyze the potential of the extract of *M. sanguineum* as a functional food or therapeutic agent. Five flavonoids (epicatechin gallate, epicatechin, rutin, epigallocatechin and quercetin) and two phenolic acids (protocatechuic acid and chlorogenic acid) have been identified from the extract (Table 4). The detected predominant phenolic compound was epicatechin gallate (ECG) (256.14 ± 18.42 µg/g DW), followed by epicatechin (22.57 ± 1.78 µg/g DW) and rutin (17.24 ± 1.52 µg/g DW). Numerous studies have demonstrated that ECG possesses anticancer efficacy, which is largely attributed to its potent antioxidant activity [31]. Moreover, a study reported that ECG inhibited atherosclerosis as it could reduce oxidative damage and apoptosis through upregulating autophagy [32]. Except for ECG, six other phenolic compounds have also shown bioactivities in protecting against several diseases [7,8]. Given these, the extract of *M. sanguineum* fruit was worth exploring as a therapeutic agent or functional food.

## 3. Materials and Methods 

### 3.1. Sample Preparation

The fresh mature fruits of *M. sanguineum* were harvested in the Lung Fu Mountain, Hong Kong, China, and identified by Dr. Xin-Sheng Qin from the College of Forestry, South China Agricultural University. The voucher specimen was preserved in the School of Public Health, Sun Yat-Sen University (No. MR-20161002-01). Collected fruits were washed, air dried, ground into particles using a grinder (RS-FS500B; Royalstar Co., Ltd., Hefei, Anhui, China), tightly sealed in airtight bags, and stored at 4 °C. The moisture of sample was measured to calculate dry weight, which was 42.5%.

### 3.2. Standards and Reagents

Trolox (6-hydroxy-2,5,7,8-tetramethylchromane-2-carboxylic acid), gallic acid, ABTS (2,2′-azino-bis(3-ethylbenothiazoline-6-sulphonic acid) diammonium salt), Folin & Ciocalteu’s phenol, and phenolic standards (such as epicatechin and epicatechin gallate) were products purchased from Sigma-Aldrich (St. Louis, MO, USA). Chromatography-grade formic acid and methanol were produced by Kermel Chemical Factory (Tianjin, China). All the other regents (such as sodium carbonate anhydrous) were of analytical grade, and were purchased from Damao Reagent Factory (Tianjin, China). 

### 3.3. Microwave-Assisted Extraction

MAE was performed using a device (X-100A; Xianghu Instrumental Company, Beijing, China), and the operating parameters (temperature, time and power) could be adjusted and controlled. The powdered *M. sanguineum* fruit (0.500 g) was extracted under different levels of ethanol concentration, solvent/material ratio, process temperature, time, and microwave power. After the centrifugation for 15 min at 4200× *g*, the supernatant of the mixture would be gathered and stored at −20 °C for further analysis within 2 days.

### 3.4. Maceration Extraction

The powdered sample of 0.500 g was immersed in 16.11 mL of 31.33% ethanol aqueous solution with stirring, and extracted for 24 h at 25 °C in a water bath shaker. After centrifugation (4200× *g*, 15 min), the collected supernatant was stored at −20 °C for further assay. 

### 3.5. Soxhlet Extraction

The Soxhlet extraction was executed according to the procedures previously reported [19]. The sample (1.000 g) was enclosed in filter paper in a Soxhlet extractor with 200 mL of 31.33% ethanol aqueous solution at 95 °C water bath for 4 h. After extraction, the obtained solution was collected and stored at −20 °C for further assay.

### 3.6. Measurement of Total Phenolic and Flavonoid Contents, and Antioxidant Capacity

The total phenolic content (TPC), total flavonoid content (TFC), and Trolox equivalent antioxidant capacity (TEAC) in extracts were measured based on the procedure previously published [19,33], and were stated as mg GAE/g DW, mg quercetin equivalent (QE)/g DW, and µmol Trolox/g DW, respectively.

### 3.7. Experimental Design and Statistical Analysis

The influences of 5 experimental factors on phenol yield from *M. sanguineum* fruit were evaluated individually. Then, 3 dominant factors that influenced the phenol yield would be selected to study their interactions in following RSM by Design Expert 8.0.6 (Stat-Ease Inc., Minneapolis, MN, USA). The three-variable–five-level CCD matrix involving 20 runs was carried out. All the assays were repeated in triplicate, and experimental data were shown as mean value ± standard deviation (SD). 

The variation of TPC values (Y) versus the 3 dominant variables (X_1_, X_2_ and X_3_) were fitted into a response surface model and expressed by the following equation:

Y = β_0_ + ∑β_i_X_i_ + ∑β_ii_X_i_^2^ + ∑β_ij_X_i_X_j_. (2)

In the equation, β_i_, β_ii_, β_ij_ and β_0_ stand for the coefficients of the linear, quadratic, interactive and constant terms, respectively. Analysis of variance (ANOVA) and the verification experiment were carried out to identify the adequacy of the response surface model. Besides, the three-dimensional surface plots were employed to visualize the interactions of independent variables on the TPC value. 

### 3.8. UPLC-MS/MS Assay

LC–MS/MS system (4000 QTRAP; AB Sciex, Framingham, MA, USA) and HSS T3 column (internal diameter: 2.1 mm, column length: 100 mm, particle size: 1.8 µm; Acquity UPLC; Waters, Milford, MA, USA) were employed with an injection volume of 2 µL to determine and quantify phenolic compounds in the extracts obtained under optimal extraction conditions. The UPLC conditions were as follows: mobile phase A of 0.1% formic acid in water; mobile phase B of methanol; temperature of 40 °C; flow rate of 300 µL/min; elution gradient of 15% B (2 min), 15% to 30% B (6 min), 30% to 80% B (7 min), 80% B (2.5 min), 15% B (2 min). The multiple reaction monitoring was used, and parameters of MS were programmed as follows: electrospray ionisation (ESI) source in negative ion mode; curtain gas of 10 psig; temperature of 550 °C; ionspray voltage of −4500 V; ion source gas 1 and 2 both of 20 psig. Finally, the parent ions (*m*/*z*, [M − N]^−^), product ions (*m*/*z*), peak retention times (t_R_, min) and peak areas of samples were compared with those of phenol standards to verify and quantify phenolic compounds in the extracts, and the contents were expressed as µg/g DW.

## 4. Conclusions

The experimental results showed the potential of MAE to extract phenolic compounds, especially epicatechin gallate from the fruit of *M. sanguineum*. A three-variable–five-level CCD of 20 combinations was successfully applied for the optimization of the MAE technique by RSM, and a second-order polynomial regression model with high reliability and validity was obtained. The best extraction conditions were as follows: 31.33% ethanol, 32.21 mL/g, 45 min, 52.24 °C and 500 W, and the maximum TPC value was 39.02 ± 0.73 mg GAE/g DW. In addition, MAE remarkably increased TPC, TEAC and TFC values of the extract compared with maceration or Soxhlet extraction in a shorter time. Besides, the MAE markedly reduced the use of organic solvents and extraction duration in comparison with the Soxhlet extraction. Taken together, the present work contributed to the exploration of *M. sanguineum* fruit as a good source of natural phenolic compounds, especially epicatechin gallate, which has multiple bioactivities and health benefits.

## Figures and Tables

**Table 1:** The central composite design, coded and actual levels of three independent variables, actual and predicted values of total phenolic content.

Run	X_1_ (Ethanol Concentration, %)	X_2_ (Solvent/Material Ratio, mL/g)	X_3_ (Temperature, °C)	Y (TPC Value, mg GAE/g DW)	
				Actual	Predicted
1	20 (−1)	40 (1)	40 (−1)	28.71	27.70
2	40 (1)	40 (1)	60 (1)	30.32	30.32
3	30 (0)	30 (0)	50 (0)	39.30	38.68
4	30 (0)	46.82 (1.68)	50 (0)	30.33	30.50
5	40 (1)	20 (−1)	60 (1)	28.11	29.60
6	20 (−1)	20 (-1)	60 (1)	26.93	27.47
7	30 (0)	30 (0)	50 (0)	39.42	38.68
8	40 (1)	20 (−1)	40 (−1)	23.11	22.42
9	30 (0)	30 (0)	66.82 (1.68)	32.63	30.96
10	30 (0)	30 (0)	50 (0)	38.17	38.68
11	20 (−1)	40 (1)	60 (1)	28.11	29.28
12	13.18 (−1.68)	30 (0)	50 (0)	27.72	27.25
13	40 (1)	40 (1)	40 (−1)	29.75	29.69
14	30 (0)	30 (0)	50 (0)	38.65	38.68
15	30 (0)	30 (0)	33.18 (−1.68)	22.61	23.60
16	20 (−1)	20 (−1)	40 (−1)	18.87	19.35
17	30 (0)	30 (0)	50 (0)	37.51	38.68
18	30 (0)	13.18 (−1.68)	50 (0)	23.72	22.87
19	46.82 (1.68)	30 (0)	50 (0)	30.91	30.70
20	30 (0)	30 (0)	50 (0)	38.90	38.68

**Table 2:** Analyses of variance of the regression model.

Source	Sum of Squares	df	Mean Square	*F* Value	*p* Value
Model	726.77	9	80.75	62.17	<0.0001
X_1_	14.42	1	14.42	11.10	0.0076
X_2_	70.31	1	70.31	54.13	<0.0001
X_3_	65.38	1	65.38	50.34	<0.0001
X_1_X_2_	0.59	1	0.59	0.45	0.5161
X_1_X_3_	0.45	1	0.45	0.34	0.5707
X_2_X_3_	21.42	1	21.42	16.49	0.0023
X_1_^2^	169.56	1	169.56	130.54	<0.0001
X_2_^2^	259.05	1	259.05	199.44	<0.0001
X_3_^2^	233.98	1	233.98	180.14	<0.0001
Residual	12.99	10	1.30		
Lack of Fit	10.38	5	2.08	3.98	0.0778
Pure Error	2.61	5	0.52		
Cor. Total	739.76	19			
R^2^	0.9824				
Adjusted R^2^	0.9666				

df: degree of freedom.

**Table 3:** The comparison of microwave-assisted extraction with maceration and Soxhlet extraction.

Extraction Methods	Ethanol Concentration (%)	Time	Temperature (°C)	TPC (mg GAE/g DW)	TEAC (μmol Trolox/g DW)	TFC (mg QE/g DW)
Maceration extraction	31.33	24 h	25	25.79 ± 1.03	380.66 ± 1.09	1.11 ± 0.28
Soxhlet extraction	31.33	4 h	95	18.40 ± 1.34	309.10 ± 1.32	1.19 ± 0.23
MAE	31.33	45 min	52.24	39.02 ± 0.73	480.58 ± 1.23	1.33 ± 0.31

TEAC: Trolox equivalent antioxidant capacity; TFC: total flavonoid content; QE: quercetin equivalent.

**Table 4:** The phenolic compounds in extract of *M. sanguineum* fruit.

Phenolic Compounds	Classification	Retention Time (*t*_R_, min)	Parent Ion (*m/z*, [M − H]^−^)	Product Ion (*m/z*)	Contents (µg/g DW)
Epicatechin gallate	Flavonoid	6.87	441	169	256.14 ± 18.42
Epicatechin	Flavonoid	5.4	289	203	22.57 ± 1.78
Rutin	Flavonoid	9.67	609	300	17.24 ± 1.52
Epigallocatechin	Flavonoid	3.03	305	137	7.84 ± 0.67
Protocatechuic acid	Phenolic acid	3.09	152.9	107.8	0.74 ± 0.14
Chlorogenic acid	Phenolic acid	4.13	353	191	0.65 ± 0.08
Quercetin	Flavonoid	11.8	301	179	0.35 ± 0.02
